# Deployment of national criteria for prevention of infection in surgical site in Ponta Grossa, Parana, Brazil

**DOI:** 10.1186/1753-6561-5-S6-P197

**Published:** 2011-06-29

**Authors:** M Gaspar, CLD Silva, CRB Grden, CAS Ito, L Bail, AM Pipino, WDDO Collares

**Affiliations:** 1 Departamento de Enfermagem e Saúde Pública, Ponta Grossa, Brazil; 2 Departamento de farmácia, Universidade Estadual de Ponta Grossa – Parana, Ponta Grossa, Brazil; 3 Hospital Regional de Ponta Grossa, Ponta Grossa, Brazil; 4 Universidade Estadual de Ponta Grossa – Parana, Ponta Grossa, Brazil

## Introduction / objectives

The Surgical Site Infection (SSI) is one of the chief aggravation related to health care in Brazil, occupying the third position among all infections with a prevalence of 14% to 16% of those found in hospitalized patients. This is a prospective, qualitative and descriptive study whose aim was to implement preventive measures to control SSI at a Public Hospital, Ponta Grossa-PR, 2010.

## Methods

A transposition of the " National Criteria of Infections related to prevention of SSI" had been carried out, ruled by the National Health Surveillance Agency (ANVISA, 2009) to the 15 surgeons and 12 nurses from the operating room and surgical clinic, as well as the application of a questionnaire with the purpose of verifying the concept of the team about the packages of measures, analyzed by the content of Bardin.

## Results

The results showed that 18 (67%) of the participants had a shortage of knowledge concerning the packages of measures. Regarding their conceptions, the results were organized into two categories: Permanent Health Education, aimed at minimizing risks and Implementation of Preventive Measures. Thus, this study has provided subsidies to systematize the preventive measures contemplating from pre to post-operative period, ensuring maximum integrity, recovery and mitigation of risks to patient health.

## Conclusion

When you think the reorganization of health care practice, this should be done on new bases and criteria to replace the traditional model of care.

## Disclosure of interest

None declared.

